# Long-Term Exposure to Air Pollutants and Cancer Mortality: A Meta-Analysis of Cohort Studies

**DOI:** 10.3390/ijerph15112608

**Published:** 2018-11-21

**Authors:** Hong-Bae Kim, Jae-Yong Shim, Byoungjin Park, Yong-Jae Lee

**Affiliations:** 1 Department of Family Medicine, MyongJi Hospital, Hanyang University Medical Center, 14-55 Hwasu-ro, Deokyang-gu, Goyang, Gyeonggi-do 10475, Korea; hongbai96@naver.com; 2 Department of Medicine, Graduate School of Yonsei University, 50-1 Yonsei-ro, Seodaemoon-gu, Seoul 03722, Korea; hope@yuhs.ac (J.-Y.S.); BJPARK96@yuhs.ac (B.P.); 3 Department of Family Medicine, Severance Hospital, Yonsei University College of Medicine, 50-1 Yonsei-ro, Seodaemoon-gu, Seoul 03722, Korea; 4 Department of Family Medicine, Yongin Severance Hospital, 225 Gumhak-ro, Cheoin-gu, Yongin, Gyeonggi-do 17046, Korea; 5 Department of Family Medicine, Gangnam Severance Hospital, 211 UnJu-ro, Seoul 06273, Korea

**Keywords:** air pollutants, cancer mortality, cohort study, meta-analysis

## Abstract

The aim of this study was to examine the relationship between main air pollutants and all cancer mortality by performing a meta-analysis. We searched PubMed, EMBASE (a biomedical and pharmacological bibliographic database of published literature produced by Elsevier), and the reference lists of other reviews until April 2018. A random-effects model was employed to analyze the meta-estimates of each pollutant. A total of 30 cohort studies were included in the final analysis. Overall risk estimates of cancer mortality for 10 µg/m^3^ per increase of particulate matter (PM)_2.5_, PM_10_, and NO_2_ were 1.17 (95% confidence interval (CI): 1.11–1.24), 1.09 (95% CI: 1.04–1.14), and 1.06 (95% CI: 1.02–1.10), respectively. With respect to the type of cancer, significant hazardous influences of PM_2.5_ were noticed for lung cancer mortality and non-lung cancer mortality including liver cancer, colorectal cancer, bladder cancer, and kidney cancer, respectively, while PM_10_ had harmful effects on mortality from lung cancer, pancreas cancer, and larynx cancer. Our meta-analysis of cohort studies indicates that exposure to the main air pollutants is associated with increased mortality from all cancers.

## 1. Introduction

The global level of particulate matter <2.5 μm in size (PM_2.5_) rose by 11.2% from 1990 (39.7 μg/m³) to 2015 (44.2 μg/m³), and exposure to PM_2.5_ was the fifth most common cause of death in 2015 globally, resulting in the deaths of 4.2 million people [1]. Ambient air pollutants were recently classified as lung carcinogens by the International Agency for Research on Cancer of the World Health Organization (WHO) and are considered as “the most extensive environmental carcinogens” [2].

To date, three meta-analyses [3,4,5] have examined the association between air pollution and lung cancer mortality; in them, a 10 µg/m^3^ increase in PM_2.5_ levels increased the risk of and mortality from cancer by 9%, 9%, and 7%, respectively. However, these three meta-analyses used both incidence and mortality data of lung cancer. Importantly, there is a difference between cancer incidence and mortality, because not all patients suffering from cancer will die from the disease [6]. Recent prospective cohort data collected from 623,048 participants over 22 years showed that a 4.4 μg/m^3^ increase in PM_2.5_ levels increased kidney and bladder cancer mortality rates by 14% and 13%, respectively [7]. Nitrogen dioxide (NO_2_) was also positively linked to increased mortality from colorectal cancer in this study (hazard ratio (HR) per 6.5 parts per billion (ppb): 1.06; 95% confidence interval (CI): 1.02–1.10).

At present, there are no reported quantitative meta-analyses on the association between ambient air pollution and mortality from all types of cancers. The current study addressed this gap by performing a meta-analysis of 30 cohort studies, as well as various subgroup analyses of the factors that might influence the results.

## 2. Materials and Methods

### 2.1. Data Sources and Searches

We searched PubMed and EMBASE (a biomedical and pharmacological bibliographic database of published literature produced by Elsevier) from October 1958 to April 2018 using common keywords related to air pollutants and cancer mortality. The keywords were “air pollution”, “air pollutants”, “particulate matter”, “nitrogen dioxide”, “sulfur dioxide”, and “ozone” for exposure factors and “cancer”, “malignancy”, and “carcinoma” for outcome factors. Additionally, we inspected the bibliographies of related articles and reviews to identify additional pertinent data.

### 2.2. Study Selection and Eligibility

We included observational articles that met the following criteria: (1) a prospective or retrospective cohort study; (2) examined the association between air pollution and mortality from any type of cancer; and (3) reported outcome measures with adjusted relative risk (RR) and 95% CI. When two or more analyses contained duplicated data or used the same participants, we included the more comprehensive analysis. We excluded the following: (1) in vivo and in vitro studies; (2) case reports, review articles, and letters; (3) studies on cancer incidence but not mortality; (4) studies with inconvertible data; and (5) studies assessing indoor, occupational, or accidental exposures to pollutants.

Using the selection criteria, three authors (H.B.K., J.Y.S., and B.P.) independently assessed the eligibility of the retrieved articles. Any disagreements among the evaluators were resolved by discussion with the help of a fourth author (Y.J.L.).

### 2.3. Data Extraction 

Two authors (H.B.K. and B.P.) independently extracted the study characteristics from the eligible articles, which were then reviewed by a third author (Y.J.L). The extracted data included the name of the first author, publication year, type of cohort study, year in which the participants were enrolled, location of the study, means of quantifying exposure (e.g., degree of exposure, mean concentration of pollutants), number of cases, type and stage of cancer, adjusted confounding variables, and adjusted RR ratios and 95% CI.

### 2.4. Assessment of Methodological Quality

We used the Newcastle–Ottawa Scale (NOS) [8] to estimate the methodological quality of the studies included in our meta-analysis. The NOS is comprised of three subscales (selection of studies, comparability, and exposure), and its scores range from 0–9. There is no established cut-off point for high versus low quality; hence, we rated studies with higher than average scores as high-quality and analyzed all studies despite their score.

### 2.5. Main and Subgroup Analyses

The main analysis examined the association between long-term exposure to air pollutants and cancer mortality. Subgroup analyses assessed the effect of the following factors on cancer mortality: type of air pollutant, gender, geographical region, duration of cohort study, mean pollutant concentration according to WHO guidelines, type of cancer, stage of cancer, number of participants, methodological quality, and smoking status. Subgroup analyses were conducted separately for the two pollutants that most significantly impacted cancer mortality.

### 2.6. Statistical Analyses

Because most exposure-response meta-analyses consider the relationship between air pollution and disease mortality to be linear [9,10], our protocol also included standardized increments: a 10 μg/m^3^ increase in exposure to PM_2.5_; particulate matter <10 μm in size (PM_10_); NO_2_, nitrogen oxides (NO_x_), and sulfur dioxide (SO_2_); and a 10 ppb increase in exposure to ozone (O_3_). We recalculated the RR for the standardized increment for each pollutant by applying the following formula [11]:

$$RR_{Standardized}=e^{\left(\frac{\ln\left(RR_{Origin}\right)}{Increment_{Origin}}\;\times \;Increment_{Standardized}\right)}\;$$

where RR is the relative risk and ln is the log to the base e. If the RR was presented on a continuous scale as an interquartile range (IQR), we used the increment in IQR instead of the increments noted above. 

To evaluate the association between air pollutants and cancer mortality, a pooled RR ratio and 95% CI was calculated from the adjusted RR ratio and 95% CI in each study. To test heterogeneity across studies, we used the Higgins I^2^ test to determine the percentage of total variation [12]. I^2^ was computed as follows:

I^2^ = 100% × (*Q* − *df*)/*Q*

where *Q* is Cochran’s heterogeneity statistic and *df* indicates the degrees of freedom. I^2^ values ranged from 0% (no observed heterogeneity) to 100% (maximal heterogeneity), with values >50% indicating substantial heterogeneity [12]. A random-effects model based on the DerSimonian and Laird method was used for calculating the overall RR and 95% CI values, because populations and methodologies differed among the studies [13].

We assessed publication bias using Begg’s funnel plot and Egger’s test [14]. When bias was present, the funnel plot showed asymmetry or Egger’s test had a *p*-value <0.05. We used Stata SE software, version 13.1 (StataCorp, College Station, TX, USA) for the statistical analyses.

## 3. Results

### 3.1. Eligible Studies

**Figure 1:**
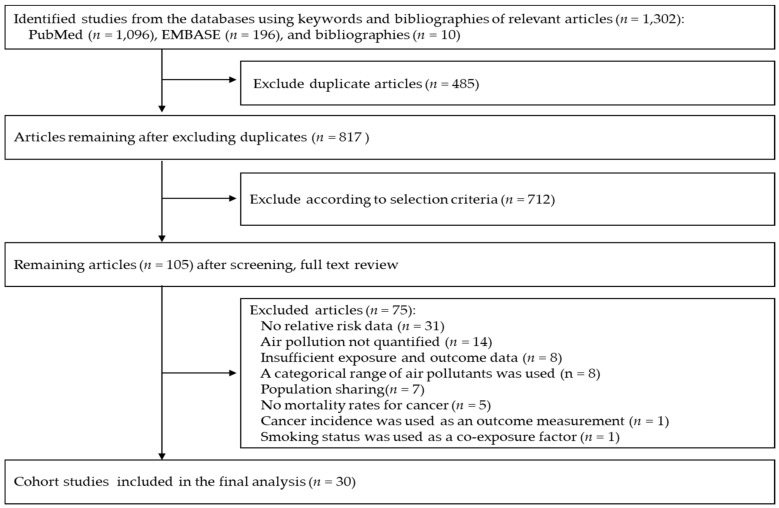
Flow diagram for identification of relevant studies.

The abstracts of a total of 1302 articles were identified in the initial investigation of two databases and by hand-searching relevant bibliographies. After excluding 485 duplicated articles, two of the authors independently surveyed the eligibility of all studies and excluded an additional 712 articles that did not meet the predetermined inclusion criteria (Figure 1). Finally, the full texts of the remaining 105 articles were inspected, of which 75 articles were excluded for the following reasons: no RR data (n = 31), air pollution not quantified (n = 14), insufficient exposure and outcome data (n = 8), a categorical range of air pollutants was used (n = 8), population sharing (n = 7), no mortality rates for cancer (n = 5), cancer incidence was used as an outcome measurement (n = 1), and smoking status was used as a co-exposure factor (n = 1). The remaining 30 cohort studies were included in the meta-analysis [7,15,16,17,18,19,20,21,22,23,24,25,26,27,28,29,30,31,32,33,34,35,36,37,38,39,40,41,42,43]. All cohort studies were prospective except the study by Ancona et al. [35], which was retrospective.

### 3.2. Characteristics of Studies Included in the Final Analysis

Table 1 shows the general characteristics of the 30 cohort studies included in our meta-analysis. All studies were published between 1999 and 2017 and together comprised >36,077,332 participants. In studies reporting age, the mean age of the participants was 57.3 years (range: 0–120 years). Regarding the type of cancer, most of them concerned lung cancer, while some of them involved all types. Mostly, the selected studies were conducted in the United States (n = 10), the Netherlands (n = 3), and China (n = 3). Adjusted variables of each study were presented in Table A1.

Ten studies used fixed-site monitor measurements for the exposure assessment method, while 17 studies used modeling-based assessment methods such as land-use regression or air dispersion models. All studies except three [29,31,35] were funded by public/governmental organizations or independent scientific foundations. The NOS scores of the studies ranged from 5 to 9; the average score was 7.7. The number of high-quality studies (NOS score ≥ 8) was 21. Data were extracted from the general population in all studies except four, which were conducted on breast cancer patients [29], lung cancer patients [37], patients with myocardial infarction [40], and liver cancer patients [43], respectively.

### 3.3. Overall Meta-Estimates and Publication Bias

**Figure 2:**
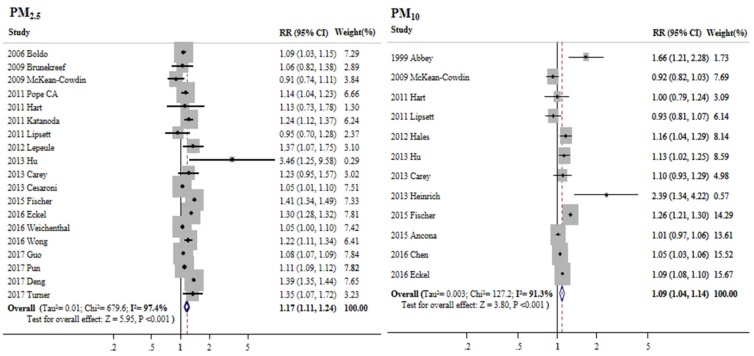
Mortality from cancer according to long-term exposure to particulate matter (PM) in a random-effects meta-analysis of observational studies. RR, relative risk; CI, confidence interval (RR and 95% CI are for a 10 μg/m^3^ increase in PM_2.5_ and PM_10_).

**Figure 3:**
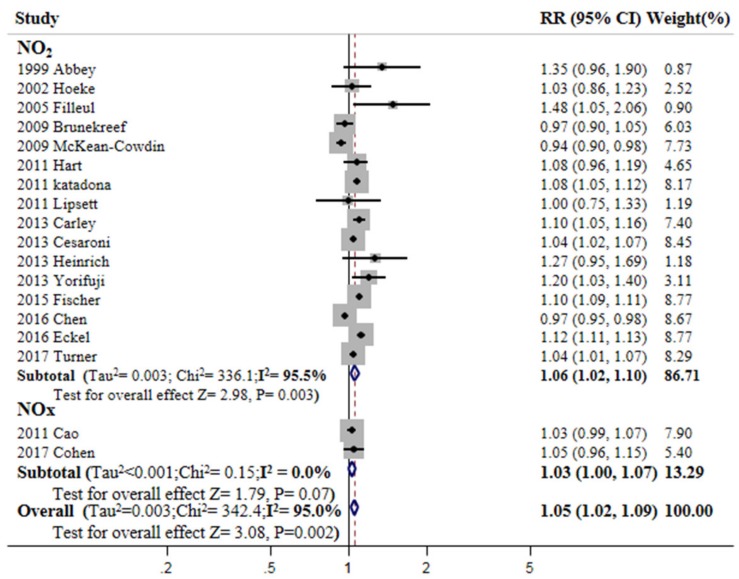
Mortality from cancer according to long-term exposure to nitrogen dioxide (NO_2_) and nitrogen oxides (NO_x_) in a random-effects meta-analysis of observational studies. RR, relative risk; CI, confidence interval (RR and 95% CI are for a 10 μg/m^3^ increase in NO_2_ and NO_x_).

All-cancer mortality significantly correlated with long-term exposure to PM_2.5_ (RR: 1.17; 95% CI: 1.11–1.24; I^2^: 97.4%), PM_10_ (RR: 1.09; 95% CI: 1.04–1.14; I^2^: 45.7%) (Figure 2), and NO_2_ (RR: 1.06; 95% CI: 1.02–1.10; I^2^: 95.5%) (Figure 3). Significant, although less strong, mortality associations were also observed for NO_x_ (RR: 1.03; 95% CI: 1.00–1.07; I^2^: 0.0%) and SO_2_ (RR: 1.03; 95% CI: 1.00–1.05; I^2^: 56.6%). Pooled data for NO_2_ and NO_x_ indicated that air pollutants composed of nitrogen compounds significantly increased the risk of cancer mortality (RR: 1.05; 95% CI: 1.02–1.09; I^2^: 95.0%). Exposure to O_3_ reduced the risk estimate, albeit not to a significant extent (RR: 0.98; 95% CI: 0.90–1.07; I^2^: 74.5%; not shown in figure). In Table A2, a stratified analysis showed no publication bias in terms of the results for PM_2.5_, PM_10_, and NO_2_ (Egger’s test for asymmetry: *p* = 0.40, 0.68, and 0.41, respectively; Begg’s funnel plots were all symmetrical).

### 3.4. Subgroup Analyses of the Association between PM_2.5_ and Cancer Mortality

The significant relationship between PM_2.5_ and cancer mortality was very similar in the subgroup analyses stratified by gender, geographical region, follow-up period, mean levels of pollutant concentration, stage of cancer, number of participants, methodological quality, and smoking status.

As shown in Table 2, long-term exposure to PM_2.5_ increased mortality from liver cancer, colorectal cancer, bladder cancer, and kidney cancer, as well as mortality from lung cancer. There was a similar association between PM_2.5_ and mortality from non-lung cancer (RR: 1.16, 95% CI: 1.04–1.30) when compared with mortality from lung cancer (RR: 1.14, 95% CI: 1.07–1.21). In addition, early stage cancer was more prominent in relation to air pollution and cancer mortality (RR: 1.81, 95% CI: 1.63–2.01 for localized state; RR: 1.47, 95% CI: 1.36–1.59 for regional state; and RR: 1.17, 95% CI: 1.05–1.30, for metastatic state, respectively).

### 3.5. Subgroup Analyses of the Association between PM_10_ and Cancer Mortality

Long-term exposure to PM_10_ significantly correlated with cancer mortality in subgroup analyses stratified by mean pollutant concentration, cancer stage, methodological quality, and smoking status. As shown in Table 2, it increased the mortality rate in pancreas cancer, larynx cancer, and lung cancer. However, PM_10_ was not related to mortality from cancers other than lung cancer, in contrast to PM_2.5_. Similar to PM_2.5_, PM_10_ best correlated with mortality in early-stage cancer.

PM_10_, unlike PM_2.5_, did not adversely affect mortality rates in men, women, patients in Europe, patients with follow-up periods <10 years, and a small study size.

## 4. Discussion

Our meta-analysis of 30 cohort studies involved >1.0 million cases in 14 countries and hence provided sufficient statistical power. It showed that ambient air pollution significantly correlated with cancer mortality in analyses including all participants, as well as those stratified for various factors. Among the pollutants examined, PM_2.5_, PM_10_, or NO_2_ were most strongly associated with cancer mortality, whereas O_3_ was not significantly associated.

The deleterious effects of air pollution on survival were not limited to the lungs, but also included non-lung organs, especially in cancer patients exposed to PM_2.5_. Evidence from several in vivo studies suggests that particulate pollutants can travel to the liver, kidneys, and brain [44,45,46]. Our study indicates that air pollution is more strongly linked to cancer mortality in early-stage patients than those in later stages. Although many clinicians presume that the opposite is true, current research shows that patients in earlier stages of cancer may require more education regarding air pollution exposure prevention.

How air pollution increases cancer mortality rates is unclear, but two mechanisms have been proposed. The first mechanism involves DNA damage due to oxidative stress. Reactive oxygen species cause oxidative stress and are generated in response to PM [47]. Nitrogen pollutants can exacerbate the effects of oxidative stress on the progression of breast, prostate, colorectal, cervical, and other cancers [48]. Exposure to SO₂ is extremely harmful, as it induces oxidative stress in many organs [49]. Undue oxidative stress in cancer cells may seriously affect survival outcomes by promoting cell proliferation, genetic instability, and mutations [50]. In a prospective cohort study from the United States that included 30,239 Caucasian and African-American participants, there was a significant association between an oxidative stress and cancer mortality [51].

The second mechanism involves inflammation. In an in vitro study, inhaled gaseous and particulate pollutants increased the production of proinflammatory cytokines such as interleukin (IL)-6 and IL-8 [52]. In a cohort panel study conducted in the United States, exposure to NO_x_ and PM increased plasma IL-6 levels over a 12-week period [53]. The poor prognosis of gastric cancer and non-Hodgkin’s lymphoma has been linked to excessive amounts of the proinflammatory cytokines tumor necrosis factor and IL-1, respectively [54,55]. Furthermore, the production of tumor-associated macrophages, which occurs during the inflammatory reaction, is a sign of an exacerbated cancer state [56]. Thus, inflammation caused by exposure to air pollution may result in cancer mortality.

Unlike the other pollutants in our study, O_3_ did not significantly impact lung cancer and brain cancer survival. Similarly, in the meta-analysis conducted by Atkinson et al. on lung cancer only [57], there was no association between long-term exposure to O_3_ and lung cancer mortality (RR: 0.95; 95% CI: 0.83–1.08; I^2:^: 55%). Nonetheless, evaluating this relationship is challenging. O_3_ is comprised of a combination of noxious air elements termed the “photochemical cocktail”, and its mechanisms of formation and destruction differ from those of other pollutants [58].

The key strengths of our meta-analysis are its inclusion of all cancer types, its separation of cancer mortality from cancer incidence, and its coverage of more countries and cases than previous studies. It also included more factors in its subgroup analyses than did the three previous meta-analyses that assessed the association between air pollution and lung cancer risk [3,4,5]. Furthermore, unlike previous studies, it examined the impact of air pollution on non-lung cancer mortality as well as lung cancer mortality. Overall, it provided the most comprehensive information to date on the mortality risk of cancer patients exposed to the main air pollutants.

The limitations of the current study include (1) no distinction between urban and rural areas; (2) considerable heterogeneity as indicated by the Higgins I^2^ values; (3) no information about indoor air pollution caused by heating, cooking, and passive smoking; (4) inclusion of only one or two studies in most cancer subgroups (lung and breast cancers were the exceptions); and (5) no data on confounding factors such as physical activity, X-ray testing, and radon exposure [59].

## 5. Conclusions

Our data showing a robust association between air pollution and all-cancer mortality have important implications for public health. This association applied to almost all of the pollutants examined in the study and was strongest for particulate pollutants in the regions wherein their mean concentrations were below standard levels. Similarly, a recent cohort study in the United States with >60 million participants found that exposure to PM_2.5_ increased all-cause mortality rates at concentrations below the present national limits [60]. Hence, rigorous environmental health policies are needed to keep air pollution levels, and consequently cancer mortality rates, as low as possible. Additionally, our results show that different types of PM increase the mortality rates for different types of non-lung cancers (PM_2.5_: liver, colorectal, bladder, and kidney; PM_10_: pancreas and larynx); hence, they may act via different mechanisms. Future research should focus on the association between certain types of pollutants and mortality from organ- and type-specific cancers.

## Figures and Tables

**Table 1:** General characteristics of the cohort studies included in the final analysis (n = 30).

References (Publication Year)	Type of Cohort Study	Country	Years Enrolled	Number of Cases	Cancer Site	Definition of Pollutant Exposure (Incremental Increase)	RR (95% CI)	Quality Assessment (Newcastle–Ottawa Stars)
Abbey et al. (1999) [15]	Prospective	USA	1977–1992	29 cases	Lung	PM_10_ 24.08 µg/m^3^ increase	3.36 (1.57–7.19)	8
Hoek et al. (2002) [16]	Prospective	Netherlands	1986–1994	244 cases	Non-lung	NO_2_ 30 µg/m^3^ increase	1.08 (0.63–1.85)	9
Nafstad et al. (2004) [17]	Prospective	Norway	1972–1998	382 cases	Lung	NO_x_ 10 µg/m^3^ increase	1.11 (1.03–1.19)	8
Filleul et al. (2005) [18]	Prospective	France	1974–2000	178 cases	Lung	NO_2_ 10 µg/m^3^ increase	1.48 (1.05–2.06)	9
Boldo et al. (2006) [19]	Prospective	Spain	1999–2003	1901 cases	Lung	PM_2.5_ 15 µg/m^3^ increase	1.14 (1.04–1.23)	5
Brunekreef et al. (2009) [20]	Prospective	Netherlands	1987–1996	1935 cases	Lung	PM_2.5_ 10 µg/m^3^ increase	1.06 (0.82–1.38)	8
McKean-Cowdin et al. (2009) [21]	Prospective	USA	1982–1988	1284 cases	Brain	PM_2.5_ 10 µg/m^3^ increase	0.91 (0.74–1.11)	8
Cao et al. (2010) [22]	Prospective	China	1991–2000	624 cases	Lung	SO_2_ 10 µg/m^3^ increase	1.04 (1.02–1.06)	8
Poppe CA et al. (2011) [23]	Prospective	USA	1983–1988	3194 cases	Lung	PM_2.5_ 10 µg/m^3^ increase	1.14 (1.04–1.23)	8
Hart et al. (2011) [24]	Prospective	USA	1985–2000	800 cases	Lung	PM_2.5_ 4 µg/m^3^ increase	1.02 (0.95–1.10)	6
Katanoda et al. (2011) [25]	Prospective	Japan	1983–1992	518 cases	Lung	PM_2.5_ 10 µg/m^3^ increase	1.24(1.12–1.37)	8
Lipsett et al. (2011) [26]	Prospective	USA	1996–2005	234 cases	Lung	PM_2.5_ 10 µg/m^3^ increase	0.95 (0.70–1.28)	8
Lepeule et al. (2012) [27]	Prospective	USA	1974–2009	350 cases	Lung	PM_2.5_ 10 µg/m^3^ increase	1.37 (1.07–1.75)	9
Hales et al. (2013) [28]	Prospective	New Zealand	1996–1998	1686 cases	Lung	PM_10_ 1 µg/m^3^ increase	1.02 (1.00–1.03)	8
Hu et al. (2013) [29]	Prospective	USA	1999–2009	255,128 women	Breast	PM_10_ 10 µg/m^3^ increase	1.13 (1.02–1.25)	6
Carey et al. (2013) [30]	Prospective	United Kingdom	2003–2007	5273 cases	Lung	PM_2.5_ 1.9 µg/m^3^ increase	1.04 (0.99–1.09)	6
Cesaroni et al. (2013) [31]	Prospective	Italy	2001–2010	12,208 cases	Lung	PM_2.5_ 10 µg/m^3^ increase	1.05 (1.01–1.10)	8
Heinrich et al. (2013) [32]	Prospective	Germany	1990-2008	41 cases	Lung	PM_10_ 7 µg/m^3^ increase	1.84 (1.23–2.74)	8
Yorifuji et al. (2013) [33]	Prospective	Japan	1999–2009	116 cases	Lung	NO_2_ 10 µg/m^3^ increase	1.20(1.03–1.40)	8
Fischer et al. (2015) [34]	Prospective	Netherlands	2004–2011	53,735 cases	Lung	PM_10_ 10 µg/m^3^ increase	1.26 (1.21–1.30)	8
Ancona et al. (2015) [35]	Retrospective	Italy	2001–2010	2196 cases	All	PM_10_ 27 µg/m^3^ increase	1.04 (0.92–1.17)	8
Chen et al. (2016) [36]	Prospective	China	1998–2009	140 cases	Lung	PM_10_ 10 µg/m^3^ increase	1.05 (1.03–1.06)	9
Eckel et al. (2016) [37]	Prospective	USA	1988–2009	352,053 cases	Lung	PM_2.5_ 5.3 µg/m^3^ increase	1.15 (1.14–1.16)	7
Weichenthal et al. (2016) [38]	Prospective	Canada	1991–2009	3200 cases	Lung	PM_2.5_ 10 µg/m^3^ increase	1.05 (1.00–1.10)	7
Wong et al. (2016) [39]	Prospective	Hong Kong	1998–2011	4531 cases	All	PM_2.5_ 10 µg/m^3^ increase	1.22 (1.11–1.34)	8
Cohen et al. (2016) [40]	Prospective	Israel	1992–2013	105 cases	All	NO_x_ 10 ppb increase	1.08 (0.93–1.26)	9
Guo et al. (2017) [41]	Prospective	China	1990–2009	315,530 cases	Lung	PM_2.5_ 10 µg/m^3^ increase	1.08 (1.07–1.09)	5
Pun et al. (2017) [42]	Prospective	USA	2000–2008	255,544 cases	All	PM_2.5_ 10 µg/m^3^ increase	1.11 (1.09–1.12)	7
Deng et al. (2017) [43]	Prospective	USA	2000–2009	20,221 cases	Liver	PM_2.5_ 10 µg/m^3^ increase	1.18 (1.16–1.20)	8
Turner et al. (2017) [7]	Prospective	Canada	1982–2004	43,320 cases	Non-lung	NO2 6.5 ppb increase	1.06 (1.02–1.10)	8

Abbreviations: CI, confidence interval; NO, nitrogen oxides; PM, particulate matter; ppb, parts per billion; RR, relative risk.

**Table 2:** Particulate matter and cancer mortality in the subgroup meta-analysis of cohort studies by various factors. WHO, World Health Organization.

Subgroups	PM_2.5_			PM_10_		
	No. of Studies	Summary RR (95% CI)	I^2^ (%)	No. of Studies	Summary RR (95% CI)	I^2^ (%)
Gender						
Male only	5	1.14 (1.00, 1.29)	80.5	4	1.06 (0.93, 1.22)	69.1
Female only	6	1.13 (1.05, 1.21)	32.0	6	1.03 (0.92, 1.15)	72.3
Male and Female	16	1.18 (1.11, 1.25)	97.8	6	1.10 (1.05, 1.16)	94.9
Region						
America	11	1.18 (1.08, 1.29)	97.2	6	1.05 (1.05. 1.23)	76.5
Europe	5	1.16 (1.00, 1.35)	94.9	4	1.18 (0.99, 1.41)	95.3
Asia	3	1.17 (1.05, 1.30)	85.1	1	1.05 (1.03, 1.06)	NA
Follow-up period						
<10 years	10	1.17 (1.07, 1.27)	96.3	4	1.11 (0.96, 1.29)	89.6
≥10 years	9	1.19 (1.07, 1.32)	98.1	9	1.06 (1.03, 1.09)	82.1
Mean levels of pollutant concentration according to the WHO guideline						
Below the standard	4	1.20 (1.04, 1.39)	98.3	1	1.16 (1.04, 1.29)	NA
Above the standard	12	1.18 (1.09, 1.28)	91.1	9	1.09 (1.04, 1.15)	93.1
Types of cancer						
Lung cancer	14	1.14 (1.07, 1.21)	97.1	9	1.07 (1.03, 1.11)	83.3
Cancers other than lung cancer	5	1.16 (1.04, 1.30)	90.9	3	1.05 (0.99, 1.11)	44.1
Brain cancer	2	1.00 (0.84, 1.19)	36.1	2	0.93 (0.83, 1.03)	0.0
Lymphatic & hematopoietic cancer	2	1.06 (0.90, 1.25)	10.6	1	1.04 (0.93, 1.16)	NA
Breast cancer	3	1.60 (0.94, 2.72)	83.4	2	1.06 (0.93, 1.21)	64.6
Liver cancer	2	1.29 (1.06, 1.58)	67.8	1	1.11 (0.84, 1.46)	NA
Pancreas cancer	1	0.96 (0.91, 1.02)	NA	1	1.05 (1.04, 1.28)	NA
Larynx cancer	1	1.09 (0.66, 1.79)	NA	1	1.27 (1.06, 1.54)	NA
Stomach cancer	2	1.17 (0.83, 1.65)	73.4	1	0.99 (0.84, 1.16)	NA
Colorectal cancer	2	1.08 (1.00, 1.17)	0.0	1	0.87 (0.71, 1.07)	NA
Bladder cancer	1	1.32 (1.07, 1.60)	NA	1	1.17 (0.88, 1.57)	NA
Kidney cancer	1	1.35 (1.07, 1.72)	NA	1	1.03 (0.84, 1.26)	NA
Stage of cancer						
Localized	3	1.81 (1.63, 2.01)	74.0	2	1.20 (1.12, 1.28)	45.1
Regional	3	1.47 (1.36, 1.59)	55.2	2	1.12 (1.11, 1.13)	0.0
Metastasis	3	1.17 (1.05, 1.30)	71.2	2	1.08 (1.02, 1.14)	49.3
No. of participants						
Small (<100,000) [15,16,17,18,22,24,25,27,32,33,35,36,39,40]	5	1.22 (1.15, 1.30)	0.0	6	1.05 (0.97, 1.13)	77.0
Large (>100,000) [7,19,20,21,23,28,29,30,31,34,37,38,41,42,43]	14	1.17 (1.10, 1.24)	98.1	6	1.11 (1.02, 1.21)	92.8
Methodological quality						
Low quality (<8)	9	1.14 (1.06, 1.22)	98.1	4	1.09 (1.08, 1.10)	0.0
High quality (≥8)	10	1.20 (1.08, 1.33)	93.5	8	1.10 (1.01, 1.21)	94.2
Smoking status						
Non-smokers	3	1.14 (1.01, 1.28)	0.0	1	1.66 (1.22, 2.28)	NA
Ex-smokers	3	1.47 (1.17, 1.84)	51.4			
Current smokers	2	1.33 (1.20, 1.49)	0.0			

NA, not applicable; PM, particulate matter; RR, relative risk; WHO, world health organization.

## Fundings

This research received no external funding.

## Appendix A

**Table A1:** Adjusted variables of each study.

Study	Adjusted Variables
Abbey et al. (1999) [15]	Education, smoking status, and alcohol use
Hoek et al. (2002) [16]	Age, sex, smoking status, education, occupation, SEP, BMI, alcohol consumption, total fat intake, vegetable consumption, and fruit consumption
Nafstad et al. (2004) [17]	Age, education, smoking habits, leisure-time physical activity, occupation, and risk groups for cardiovascular diseases
Filleul et al. (2005) [18]	Age; sex; smoking habits; educational level; BMI; and occupational exposure to dust, gases, and fumes
Boldo et al. (2006) [19]	Not available
Brunekreef et al. (2009) [20]	Age, sex, and smoking status
McKean-Cowdin et al. (2009) [21]	Age, sex, race, education level, number of colds in the past year, family history of brain cancer, previous radium treatment, number of head/neck X-rays, and use of vitamins
Cao et al. (2010) [22]	Age, sex, BMI, physical activity, education, smoking status, age at starting to smoke, years smoked, cigarettes per day, alcohol intake, and hypertension
Poppe CA et al. (2011) [23]	Age, sex, smoking status, education, marital status, BMI, alcohol consumption, occupational exposures, and diet
Hart et al. (2011) [24]	Age, calendar year, decade of hire, region of residence, race, ethnicity, census region of residence, the healthy worker survivor effect, and years of work in each of the job groups
Katanoda et al. (2011) [25]	Age, sex, smoking status, pack-years, smoking status of family members living together, daily green and yellow vegetable consumption, daily fruit consumption, and use of indoor charcoal or briquette braziers for heating
Lipsett et al. (2011) [26]	Age, race, smoking status, total pack-years, BMI, marital status, alcohol consumption, second-hand smoke exposure at home, dietary fat, dietary fiber, dietary calories, physical activity, menopausal status, hormone therapy use, family history of MI or stroke, blood pressure medication, aspirin use, and contextual variables (income, income inequality, education, population size, racial composition, and unemployment)
Lepeule et al. (2012) [27]	Age, sex, time in the study, BMI, education, and smoking history
Hales et al. (2013) [28]	Age, sex, ethnicity, social deprivation, income, education, smoking history, and ambient temperature
Hu et al. (2013) [29]	Age, race, marital status, cancer stage, year diagnosed, education, income, and accessibility to medical resources
Carey et al. (2013) [30]	Age, sex, smoking, BMI, and education
Cesaroni et al. (2013) [31]	Sex, marital status, place of birth, education, occupation, and SEP
Heinrich et al. (2013) [32]	Educational level and smoking history
Yorifuji et al. (2013) [33]	Age, sex, smoking category, BMI, hypertension, diabetes, financial capability, and area mean income
Fischer et al. (2015) [34]	Age, sex, marital status, region of origin, standardized household income, and neighborhood social status
Ancona et al. (2015) [35]	Age, gender, education, occupation, civil status, area-based SEP index, and outdoor nitrogen dioxide (NO_2_) concentration
Chen et al. (2016) [36]	Age, gender, marital status, education, BMI, smoking status, alcohol consumption, occupational exposures, and leisure exercise
Eckel et al. (2016) [37]	Age, sex, race/ethnicity, marital status, education index, SEP, rural-urban commuting area, distance to primary interstate highway, histology at diagnosis, year of diagnosis, and initial treatment
Weichenthal et al. (2016) [38]	Age, sex, aboriginal ancestry, visible minority status, immigrant status, marital status, highest level of education, employment status, occupational classification, and household income
Wong et al. (2016) [39]	Age, gender, BMI, smoking status, exercise frequency, education level, and personal monthly expenditure
Cohen et al. (2016) [40]	Age, sex, ethnicity, SEP, obesity at baseline, and smoking status
Guo et al. (2017) [41]	None
Pun et al. (2017) [42]	Race, smoking, diabetes, BMI, alcohol consumption, asthma, and median income
Deng et al. (2017) [43]	Age, sex, race/ethnicity, marital status, SEP, RUCA, distance to primary interstate highway, month and year of diagnosis, and initial treatments
Turner et al. (2017) [7]	Age, race/ethnicity, gender, education, marital status, BMI, smoking status, passive smoking, vegetable/fruit/fiber consumption, fat consumption, alcohol consumption, industrial exposures, occupation dirtiness index, and 1990 ecological covariates

Abbreviations: BMI, body mass index; MI, myocardial infarction; RUCA, rural–urban commuting area; SEP, socio-economic position.

**Table A2:** Assessment of publication bias using Begg’s funnel plot and Egger’s test.

Air Pollutants	*p*-Value from Egger’s Test	Begg’s Funnel Plot
**PM_2.5_**	0.40	Symmetry
**PM_10_**	0.68	Symmetry
**NO_2_**	0.41	Symmetry

Abbreviations: NO_2_, nitrogen dioxide; PM, particulate matter.
